# Activation of c-Jun predicts a poor response to sorafenib in hepatocellular carcinoma: Preliminary Clinical Evidence

**DOI:** 10.1038/srep22976

**Published:** 2016-03-11

**Authors:** Wei Chen, Weikai Xiao, Kunsong Zhang, Xiaoyu Yin, Jiaming Lai, Lijian Liang, Dong Chen

**Affiliations:** 1 Department of Pancreatico-biliary Surgery, The First Affiliated Hospital, Sun Yat-sen University, Guangzhou, Guangdong Province, 510080 China; 2 Department of Breast Oncology, Sun Yat-sen University Cancer Center, State Key Laboratory of Oncology in South China, Collaborative Innovation Center for Cancer Medicine, Guangzhou, Guangdong Province, 510060 China

## Abstract

We determined the mitogen-activated protein kinase (MAPK) gene expression profile of acquired resistance in sorafenib-sensitive hepatocellular carcinoma (HCC) cells and aimed to identify c-Jun as an important molecule mediating the efficacy of sorafenib. Differences in gene expression of the MAPK signaling between untreated and sorafenib-treated HCC cell lines were investigated using real-time polymerase chain reaction array. Western blot and real-time PCR further evaluated the expression of c-Jun. Pathological specimens from 50 patients with advanced HCC were collected to measure p-c-Jun expression. Sorafenib-resistant HCC cells demonstrated greater levels of basal c-Jun mRNA and protein compared with sorafenib-sensitive HCC cells. Sorafenib activated p-c-Jun in a dose- and time-dependent manner in PLC/PRF/5 and MHCC97H cell lines. Decreased expression levels of 6 genes after sorafenib treatment suggested a robust inhibitory impact of sorafenib on MAPK signaling in HCC cells. c-Jun and p-c-Jun expression levels were inversely correlated with the efficacy of sorafenib; a high expression level of p-c-Jun was associated with resistance to sorafenib and poor overall survival in patients with clinical HCC. p-c-Jun may act as a biomarker for predicting responses of sorafenib treatment, thus advocating targeting of JNK/c-Jun signaling as an optimal therapeutic strategy in a subset of HCC.

Hepatocellular carcinoma (HCC), the most frequent (85–90%) type of primary liver cancer (PLC), is ranked as the fifth most common malignancy worldwide. Nevertheless, the mortality and incidence rates of HCC show a continuously increasing trend^1^,^2^,^3^,^4^. HCC is highly heterogeneous compared with other cancer types due to its intrinsic pathogenic diversity, molecular heterogeneity, with high multicentric occurrence, among others^2^,^3^.

Although potentially curative surgical treatments exist for HCC, the disease is often refractory to chemotherapy. Unfortunately, a higher proportion of patients with HCC are diagnosed at advanced stages of the disease when they are unsuitable for curative treatments such as surgical resection and orthotopic liver transplantation^2^,^5^,^6^,^7^,^8^. This may be attributable to the intrinsic diversity in pathogenesis, molecular heterogeneity, multicentric occurrence, and etiology of HCC^9^. The advent of therapies targeting various signaling pathways of cell proliferation and angiogenesis has improved the health care outcomes of advanced HCC to some extent^7^.

Sorafenib, an orally bioavailable, multitargeted tyrosine kinase inhibitor with proven prognostic efficacy in HCC^10^,^11^,^12^, and is the recommended first-line treatment agent in nearly 40% patients with newly diagnosed HCC^13^. However, few cases of ineffective and incomplete response have also been reported with sorafenib. The modest clinical effects produced by the drug are as follows: 2–3% objective tumor response rate, 34–43% disease stabilization rate, and nearly 3-months of survival advantage over placebo. The precise mechanisms underlying sorafenib drug resistance remain elusive. To date, there has been limited success for most of the other anti-angiogenic molecular targeting agents^12^,^13^,^14^,^15^. The molecular targets of sorafenib include vascular endothelial growth factor receptor (VEGFR)-1, VEGFR2, VEGFR3, platelet derived growth factor receptor (PDGFR)-β, c-KIT, RET, FLT-3, and RAF^16^,^17^,^18^. The drug inhibits the kinase activity of Raf, an enzyme that plays a primary role within the mitogen-activated protein kinase (MAPK) signaling pathway. The MAPK signaling pathway is comprised consists of extracellular signal-regulated protein kinase (ERKs), c-Jun N-terminal kinase (JNKs), and p38MAPKs^19^. The activation of MAPK signaling is associated with malignant transformation of hepatocytes and tumor development during HCC carcinogenesis^20^,^21^. We have demonstrated that pERK, a molecule in the MAPK pathway, may be a candidate related to the survival of patients with HCC treated with sorafenib^22^. c-Jun, a downstream target of JNK, regulates cyclin D and VEGF, suppresses p53 pathway, and causes downregulation of p21, thereby promoting tumorigenesis^19^,^20^,^21^,^22^,^23^. However, specific changes in MAPK signaling during sorafenib treatment and the possible mechanism of JNK signaling are yet to be explored. As it has now become imperative to increase the inhibitory effects of sorafenib in the treatment of HCC, an increasing number of researchers are focusing on “sorafenib resistance” and identification of potential predictive biomarkers^24^. The ability to predict treatment outcome might lead to personalizing therapy and optimizing dosage, thus maximizing efficacy and cost benefit. In addition, this would aid in improving the healthcare outcomes in patients with HCC, markedly improving the medico-economic situation.

We first investigated *MAPK* gene expression profile of acquired resistance in sorafenib-sensitive HCC cells and aimed to identify c-Jun as an important molecule mediating the efficacy of sorafenib. The predictive value of p-c-Jun in determining overall survival (OS) was also evaluated in a series of patients with HCC treated with sorafenib.

## Results

### PLC/PRF/5 was most sensitive to sorafenib

**Figure 1:**
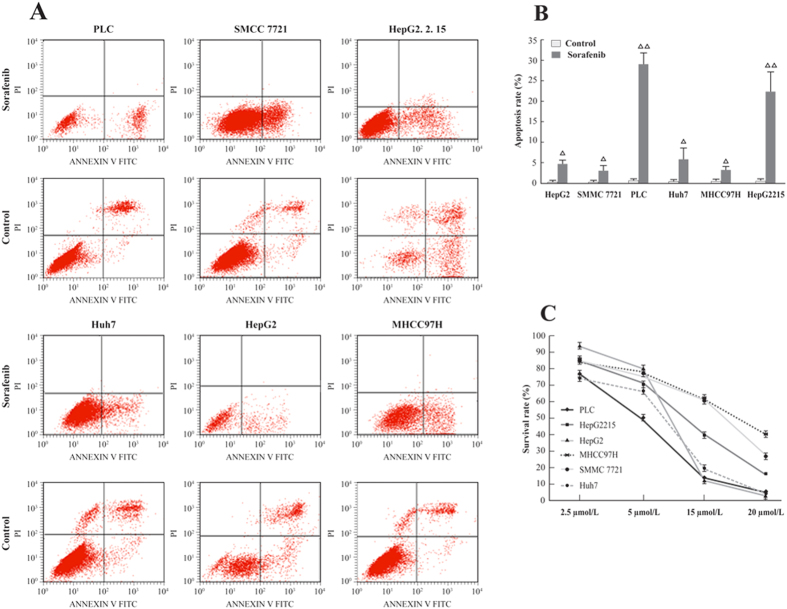
(**A**) Sorafenib induced HCC cells apoptosis; (**B**) Cell apoptosis determined by flow cytometry; (**C**) PLC/PRF/5 cells were found most sensitive to sorafenib byCCK8 cell viability test.

The molecular mechanism of the acquired resistance to sorafenib was determined by screening 6 HCC cell lines for sensitivity by using Annexin V FITC/PI stain and a CCK8 viability test. As shown in Fig. 1A,B, PLC/PRF/5 and HepG2.2.15 showed a high apoptosis probability, whereas SMCC7721 and MHCC97H were less sensitive to sorafenib. The study drug exhibited inhibitory effects in various HCC cell lines in a dose-dependent manner with the results being similar to the observations from flow cytometry (Fig. 1C). The IC50 of sorafenib was 5.25 μmol/L for PLC/PRF/5, 5.302 μmol/L for HepG2.2.15, 6.8 μmol/L for Huh7, 7.007 μmol/L for HepG2, 11.67 μmol/L for MHCC97H, and 15 μmol/L for SMCC7721. Given the observations, PLC/PRF/5 was found to be most sensitive to the effect of sorafenib.

### PCR array gene expression profiling of the MAPK pathway

**Figure 2:**
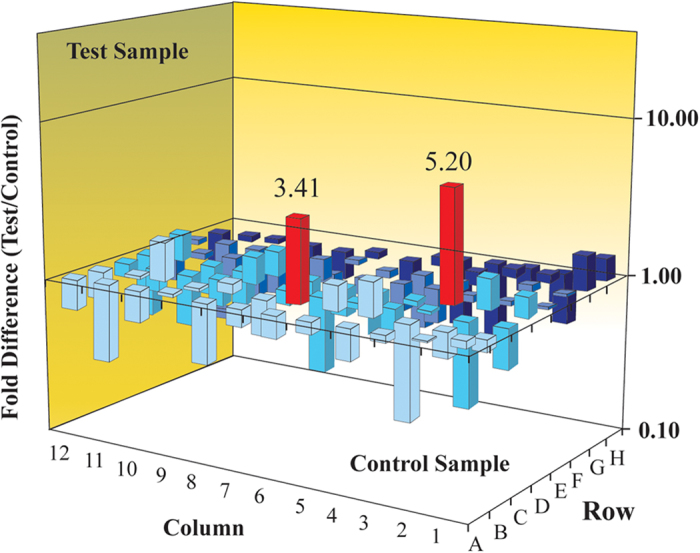
PCR array gene expression changes of MAPK signaling in sorafenib-treated PLC/PRF/5 cells. *Data analysis was based on the delta-Ct method*^46^*. Red columns represented genes upregulated fold changes higher than 2. The fold change of JUN was 5.20 (p = 5.20) and CDKN1C was 3.41 (p = 0.012).*

The results of MAPK PCR array studies demonstrated decreased expression levels of 6 genes after sorafenib treatment, suggesting a robust inhibitory impact of the drug on MAPK signaling in HCC cells (Fig. 2); however, 2 genes, *CDK6* and *JUN*, were upregulated after sorafenib treatment. Latest data revealed that c-Jun, or its upstream molecule p38MAPK, may play an important role in the mechanism of action of sorafenib on HCC cells^25^,^26^; therefore, the authors decided to further evaluate the role of the *JUN* gene in the mechanism of action of sorafenib on HCC cells in this study.

### Activation of c-Jun attenuated the inhibitory effect of sorafenib on HCC cells

**Figure 3:**
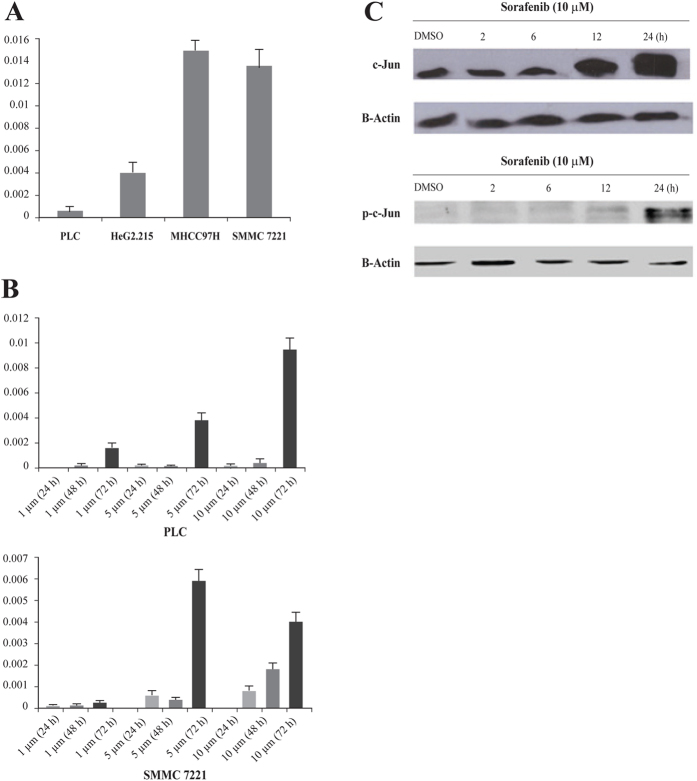
High expression of c-Jun and p-c-Jun promote resistance to sorafenib in HCC cells. (**A**) qRT-PCR analysis of the basal c-Jun expression in HCC cell lines; (**B**) Relative mRNA expression levels of c-Jun on PLC/PRF/5 and SMCC7221 cells were detected by real-time polymerase chain reaction (PCR) at transcriptional level. Data shown are the means (SD) from at least three independent experiments; (**C**) The expression of c-Jun and p-c-Jun increase after treated with sorafenib in PLC/PRF/5 cells in a time-dependent manner by western blot analysis.

c-Jun mRNA expression was lower in sorafenib-sensitive cell lines PLC/PRF/5 and HepG2.215 (Fig. 3A). In contrast, the baseline c-Jun mRNA expression was higher in sorafenib-resistant cell lines MHCC97H and SMMC7221. This implicated that a high baseline c-Jun expression may mediate a subsequent resistance to sorafenib.

Furthermore, changes in c-Jun mRNA expression during sorafenib treatment were analyzed in order to determine a potential mechanism for sorafenib resistance. Sorafenib treatment of PLC/PRF/5 and SMCC7221 cells for 72 h demonstrated dose- and time-dependent upregulation of c-Jun expression (Fig. 3B). Moreover, HCC cells with a high level of c-Jun mRNA expression remained viable, whereas others died by sorafenib-induced apoptosis. The phosphorylated (activated) form of c-Jun was quantified using phosphor-specific antibody.

Overall, both c-Jun and p-c-Jun are activated in PLC/PRF/5 cells following sorafenib treatment in a time-dependent manner at 10 μM (Fig. 3C). This suggests that c-Jun and p-c-Jun play a role in the sorafenib-induced inhibitory effect by mediating an acquired resistance.

### *p-c-Jun* immunohistological staining and overall survival in patients with HCC

**Figure 4:**
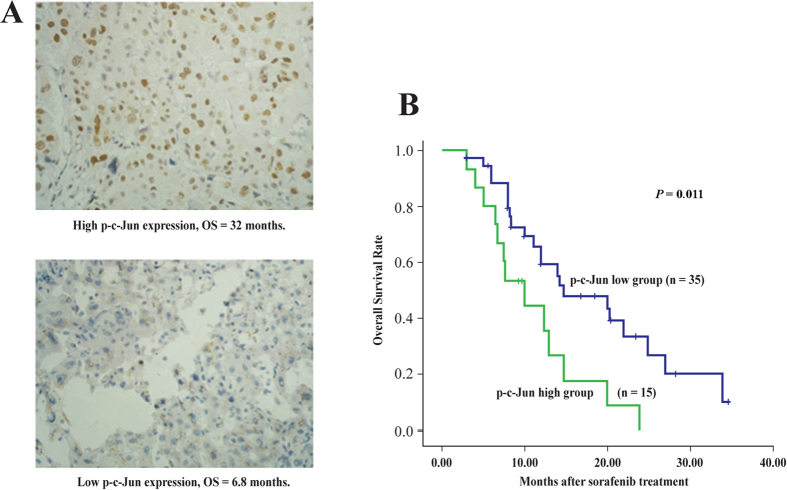
(**A**) Overall survival (OS) between patients with high and low p-c-Jun expression treated with sorafenib (**B**) Kaplan-Meier survival analysis curve.

Patient demographics (Table 1) and OS were recorded, and p-c-Jun expression was measured in 50 HCC tissue samples. p-c-Jun was localized to the tumor cell cytoplasm and nucleus. Kaplan-Meier survival analysis demonstrated that OS probability in the p-c-Jun^high^ group was much lower than that in the p-c-Jun^low^ group (Fig. 4A,B). Median OS was 10.0 months in the p-c-Jun^high^ group and 14.7 months in the p-c-Jun^low^ group (hazard ratio in p-c-Jun^high^ group: 2.328; 95% CI: 1.007-5.032; *P* < 0.05). These data indicated that a high level of p-c-Jun leads to sorafenib resistance in HCC.

## Discussion

The survival benefit from sorafenib is reported to be limited with low rates of tumor response, suggesting the existence of a drug resistance mechanism^27^,^28^. To date, potential drug resistance mechanisms have analyzed following aspects^5^: epithelial-mesenchymal transition (EMT)^29^, biological processes (tumor microenvironment, angiogenesis, inflammation, fibrosis, hypoxia, oxidative stress, autophagy, and viral reactivation)^8^,^30^,^31^,^32^, aberrant signaling activation (EFGR or mTOR signaling pathway)^31^,^33^, and, presence of cancer stem cells (CSCs)^34^. Zhai *et al.* reported that primary resistance to sorafenib might be due to genetic heterogeneity; nonetheless, activation of compensatory pathways such as PI3K/Akt and JAK/STAT pathways, tumor hypoxia, and EMT by sorafenib can lead to acquired resistance^35^. Furthermore, MAPK as the main target site of sorafenib has not been adequately explored.

PLC/PRF/5 cells were identified to be most sensitive to sorafenib treatment. The results highlight the existence of differences in the sensitivity of HCC cells to sorafenib and suggest the heterogeneity of HCC. Raf/ERK/MAPK is the primary effective signaling pathway in HCC and is the target of sorafenib therapy. However, *CDKN1C* and *p57* were found to be upregulated in the MAPK PCR array analysis of sorafenib-treated PLC/PRF/5 cells. As reported in earlier studies, *CDKN1C/p57* is involved in microRNA-mediated^36^, or, Notch signaling cascade or hepatogenesis mechanism^37^. On the basis of the results and similar observations from previous reports on the changes in c-Jun expression^25^,^26^, the authors further analyzed *JUN* gene. After treatment with sorafenib, qPCR and Western blot analyses revealed increased levels of c-Jun and p-c-Jun in HCC cells. In addition, the basal status of c-Jun mRNA expression was negatively associated with sensitivity to sorafenib. Therefore, the authors suggested that the activation of c-Jun may mediate acquired resistance and may have a protective role against sorafenib-induced cell growth inhibition. These observations were in line with those of Hagiwara *et al.*’s study, which demonstrated that elevated JNK activity in HCC was correlated with poor prognosis of sorafenib treatment^23^. Cervello *et al.* investigated sorafenib-induced alterations in global gene expression by using the Agilent 44K Human Whole Genome Oligonucleotide Microarray and Ingenuity Pathway Analysis (IPA). They observed an increase in the JNK/c-Jun signaling cascade activity after sorafenib treatment^25^. Another study screened for genes involved in sorafenib resistance using focused *in vivo* shRNA library targeting gene and revealed that p38α (upstream molecule of c-Jun) mediated acquired resistance and poor response to sorafenib through Mek-Erk and Atf2 signaling. However, the authors did not explore the changes in c-Jun in this circumstance^26^. Nguyen *et al.* suggested either ERK or p38 inhibition rescued sorafenib resistance during JNK inhibition, indicating a negative crosstalk between these signaling pathways^38^. Major MAPK cascades include ERKs, JNKs, and p38 MAPKs^39^,^40^. Few studies revealed that the molecules within the MAPK cascade themselves affect sorafenib-induced inhibition in HCC, and c-Jun may play a key role in this situation. Paradoxically, knockout of p38α in hepatocytes resulted in upregulation of the JNK/c-Jun pathway in hepatocarcinogenesis mechanism^41^. This implies that molecules within the MAPK cascade play a role in “sorafenib resistance”. However, further studies are needed to elucidate the detailed correlation between MAPK cascade and sorafenib resistance.

Hagiwara *et al.* demonstrated a significant prolongation of OS in the low p-c-Jun expression group than in the high expression group (*P* = 0.0008)^23^. Similarly, the OS probability was higher in the p-c-Jun^low^ group than in the p-c-Jun^high^ group in the present study. To recapitulate, tumors containing higher levels of p-c-Jun have shorter OS and are less responsive (or resistant) to sorafenib. Given that JNK1, the upstream molecule, is highly expressed (55%) in HCC^42^, our results which are in line with those from the previous study^23^, suggest the possibility on combined therapy with sorafenib and JNK1 inhibitor as a promising therapeutic perspective in HCC. On the contrary, only few investigations have assigned patients with sorafenib treatment to discrete prognostic groups. We have previously reported that pERK, a terminal molecule in Raf/Mek/ERK pathway, has a predictive value^22^, similar to the finding from Abou-Alfa *et al.*’s study^10^. In addition, αB-crystallin complexes were reported to be a biomarker in predicting responses to sorafenib^29^. Current analysis provides an additional candidate molecule in predicting the prognosis of patients with HCC treated with sorafenib.

It has been reported that c-Jun can translocate to the nucleus and directly bind to *Beclin 1*(a key gene of autophagy mechanism)^43^, or, the translational domain of *HRK* gene^25^. The *HRK* gene is related to the ER stress reaction, which is involved in the autophagy mechanism^44^. Combined with the evidence that activation of the JNK/c-Jun signaling cascade occurs in the autophagy mechanism of pancreatic carcinoma cells^45^, it may be assumed that JNK/c-Jun mediates acquired resistance through autophagy in HCC cells.

“Sorafenib resistance” has raised a major concern in the management of HCC and potentiation of sorafenib effects is extremely important. The current study demonstrated that activation of c-Jun may have a protective role against sorafenib in the preliminary cell studies; nonetheless, a high expression level of p-c-Jun was associated with resistance to sorafenib and poor OS in patients with clinical HCC. The results suggest that p-c-Jun may act as a biomarker for predicting responses of sorafenib treatment, thus advocating targeting of JNK/c-Jun signaling as an optimal therapeutic strategy in a subset of HCC. Further investigations will strengthen the understanding of mechanisms and effective strategies for overcoming “sorafenib resistance”.

## Materials and Methods

### Cell culture, reagents, and antibodies

Human HCC cell lines PLC/PRF/5, Huh7, HepG2, HepG2.2.15, SMMC7721, and MHCC97H were procured from the Cell Resources Bank of the Laboratory Animal Center, Sun Yat-sen University (Guangzhou, China). All the cell lines were cultured according to the manufacturer’s instructions. These were maintained in RPMI-1640 medium (Invitrogen, Gibco BRL,USA) supplemented with 10% fetal bovine serum (FBS, Invitrogen, Gibco BRL, USA), 2 mmol/L glutamine, 100 mg/L penicillin, and 100 mg/L streptomycin, and these were incubated in a humidified atmosphere of 5% CO_2_ at 37 °C. The cells were collected at 80% confluence for further experiments. For *in vitro* studies, sorafenib was dissolved in dimethyl sulfoxide (Sigma, St Louis, MO, USA) in a concentration of 10 mM. Rabbit antihuman monoclonal c-Jun antibody and rabbit antihuman monoclonal p-c-Jun antibody were obtained from Millipore (Billerica, MA, USA). Mouse antihuman monoclonal β-actin antibody was procured from Boaosen (Beijing, China).

### Flow cytometry analysis

For quantification of apoptosis, double staining was performed according to the manufacturer’s instructions by using Annexin V–fluorescein isothiocyanate (Annexin V-FITC) apoptosis kit (Franklin Lakes, NJ, USA) and propidium iodide (PI). Both attached and supernatant cells were collected, washed twice with ice-cold PBS, and resuspended in 400 μL of binding buffer. Annexin V-FITC was added to the cells and incubated for 10 min in the dark at 4 °C. PI (10 mL) was added to the tube followed by 5 min of incubation at 4 °C in the dark. After incubation, the samples were analyzed by a flow cytometry using CELL Quest software (BD), and 2.0 × 10^4^ events per sample were counted. The fraction of cell population in different quadrants was analyzed using quadrant statistics. Cells in the lower right quadrant (Annexin-V^+^/PI^−^) represented early apoptosis and those in the upper right quadrant (Annexin-V^+^/PI^+^) represented late apoptosis.

### Cellular viability test

Cells cultured in the medium containing DMSO (0.01%) were used as negative controls and the medium served as the blank control. At the end of incubation, 10 μL of Cell Counting Kit-8 (CCK-8) solution (Sigma, USA) was added into each well. Absorbance at 450 nm (A450) was measured using a microplate reader (Thermo Scientific, USA).

Growth inhibition rate (%) = [(A450 of treated group – A450 of blank control group) ÷ [(A450 of negative control group – A450 of blank control group) × 100%

The half-maximal inhibitory concentration (IC50) was then calculated from growth inhibition rates.

### Real-time polymerase chain reaction array

Total RNA was extracted from either sorafenib-treated (at a concentration of 5.25 μmmol/L, Pt group) or non-treated (DMSO, Pc group) 10^6^ PLC/PRF/5 cells using an RNeasy Mini Isolation kit (Qiangen, Germany). cDNA was synthesized from 0.5 μg RNA using an RT^2^ First Strand kit (SA Biosciences, USA) according to the manufacturer’s protocol. cDNA was applied to a SuperArray Biosciences MAPK signaling PCR array plate (including 88 genes, PAHS-061A, Supplementary Table S1) and amplification of target genes was performed with a Perkin Elmer 7300 real-time PCR system. Data analysis was based on the delta-Ct method^46^, and, the data were normalized to β-actin as recommended by the manufacturer (Qiagen, Germany).

### Quantitative real-time PCR

Total RNA was extracted with RNAiso Plus (TaKaRa, Japan). cDNA was reverse synthesized by using Prime-Script-RT reagent (Perfect Real Time) kit (TaKaRa,Japan). The subsequent real-time PCRs of c-Jun were performed using a TaKaRa SYBR-Premix Ex TaqTM (Perfect Real Time) kit (TaKaRa, Japan). Expression levels were normalized to β-actin, an internal control. The primers were synthesized by TaKaRa (China), and their sequences are listed as follows: *JUN* forward (5′-TGGGCACATCACCACTACAC-3′) and reverse (5′-AGTTGCTGAGGTTGGCGTA-3′); *β-actin* forward (5′-TGGCACCCAGCACAATGAA-3′) and reverse (5′-CTAAGTCATAGTCCGCCTAGAAGCA-3′).

### Western blot

Whole-cell extracts of PLC/PRF/5 and SK-HEP-1 cells were first washed twice with PBS and lysed in lysis buffer (KeyGEN Total Protein Extraction Kit, KeyGEN Biotech, Nanjing, China). Cytosolic fraction was collected after centrifugation at 14000 rpm at 4 °C for 15 min and total protein was quantitatively assayed with BCA Protein Assay Kit (Keygen). Protein was separated by polyacrylamide gel electrophoresis and then transferred to polyvinylidenedifluoride membranes (Millipore, MA, USA). The membranes were subsequently blocked in 5% skimmed milk for 1 h and then incubated with respective primary antibodies (anti-c-Jun and anti-p-c-Jun antibodies) in a 1:500 dilution overnight at 4 °C. The membranes were re-warmed at room temperature, and washed with PBS containing Tween-20 (PBST) for three times followed by incubation with secondary goat anti-mouse antibody in a 1:2,500 dilution for 1 h at room temperature. Proteins were visualized on the membranes using an ECL Western blotting kit (KeyGEN, Nanjing, China), and the signals were detected by Kodak X-OMAT film. The membranes were then subjected to a 15-min tripping procedure with Western blot stripping buffer (KeyGEN, Nanjing, China), followed by a 10-min wash with PBST 3 times, and subsequently analyzed forβ-actin expression.

### Predictive value of p-c-Jun in determining overall survival

A total of 50 patients (aged < 81 years; ECOG 0-1; Child-Pugh grade A and B) with advanced HCC were treated with sorafenib from December 2008 to October 2011, Table 1. Whether a patient should have the administration of sorafenib, the decision was totally taken by the multidisciplinary team (The First Affiliated Hospital, Sun Yat-sen University, Guangzhou, China), as per their clinical setting. Their pathological tumor specimens were obtained by percutaneous needle biopsy (n = 14) or surgical specimens (n = 36)^22^. The tissues were fixed in 4% paraformaldehyde, embedded in paraffin, and cut into 4-μm sections. Sorafenib was orally administered at 400 mg twice daily. The patients visited the HCC outpatient clinic every 4 weeks for an assessment of safety and tolerability. The OS rate was calculated from treatment initiation until the date of the last visit or death from any cause, with a mean follow-up time of 17.7 months.

The study protocols conformed to the ethical guidelines of the Declaration of Helsinki and were approved by the institutional review board of The First Affiliated Hospital, Sun Yat-sen University, Guangzhou, China. Signed informed consent was obtained from all patients for subsequent use of the collected tissues. The study methods were conducted in accordance with approved national and international guidelines.

### Immunohistochemical staining

To determine angiogenesis and cell proliferation in tumors, immunohistochemical analysis was performed on formalin-fixed, paraffin-embedded tumor tissues. After deparaffinization in xylene and rehydration in ethanol, the tissue sections were incubated with 3% H_2_O_2_ in methanol to quench endogenous peroxidase. Antigen retrieval was achieved by treating the tissues with citrate buffer in a pressure cooker. The sections were subsequently incubated with rabbit antihuman c-Jun antibody (dilution 1:100) or p-c-Jun antibody (dilution 1:50) at 4 °C overnight. Rabbit IgG (Biosynthesis, China) was used as a negative control. Staining was detected by adding biotinylated secondary antibodies (Maxin-Bio, Fuzhou, China), an avidin-biotin complex (Maxin-Bio), and diaminobenzidine (Maxin-Bio). The sections were then counterstained with hematoxylin. The intensities and distributions of c-Jun and p-c-Jun staining were evaluated under microscopy, respectively. For measurement of each sample, 10 visual fields were evaluated. The intensity of staining (score A) was estimated as follows: colorless: 0; buff: 1; brownish yellow: 2, and darkish brown: 3. The distribution of staining (score B) was marked as follows: no positive cells: 0; 10% positive cells: 1; 11–33% positive cells: 2; 34–66% positive cells: 3; >66%positive cells: 4. The sum of scores A and B was defined as the immunohistochemical staining level. A score of ≤2 was considered to represent low expression and a score of >3 were considered to represent high expression. All specimens were analyzed by a trained surgeon blinded to any clinical information.

### Statistical analysis

A cumulative survival curve was constructed using the Kaplan-Meier method and compared using the log-rank test. SPSS statistical software (version 15.0, SPSS Inc., Chicago, IL, United States) was used to conduct Student *t*-tests. A two-sided *P*-value of <0.05 was considered statistically significant.

## Additional Information

**How to cite this article**: Chen, W. *et al.* Activation of c-Jun predicts a poor response to sorafenib in hepatocellular carcinoma: Preliminary Clinical Evidence. *Sci. Rep.* **6**, 22976; doi: 10.1038/srep22976 (2016).

## Supplementary Material

Supplementary Table S1

## Figures and Tables

**Table 1:** Patient and tumor characteristics at baseline.

Characteristics	No. (%), N = 50s
Sex	
Male	42 (84)
Female	8 (16)
Age (years)	
<60	40 (80)
≥60	10 (20)
Etiology of liver disease	
HBV	48 (96)
Other	2 (4)
ECOG performance status	
0	30 (60)
1	20 (40)
Child-Pugh class	
A	40 (80)
B	10 (20)
Serum α-FP (ng/mL)	
<400	32 (64)
≥400	18 (36)
Portal vein invasion	
Present	12 (24)
Absent	38 (76)
Distal metastasis	
Present	26 (52)
Absent	24 (48)
Metastasis site*	
Lung	18 (36)
Bone	6 (12)
Abdomen	2 (4)
Thoracic wall	2 (4)
BCLC stage	
B	12 (24)
C	38 (76)
Previous treatment**	
None	8 (16)
Surgery	36 (72)
RFA	16 (32)
TACE	13 (26)
α-FP (ng/mL)	7134 (1.7–58 344)

*AST, aspartate aminotransferase; BCLC, Barcelona Clinic Liver Cancer; ECOG, Eastern Cooperative Oncology Group; HBV, hepatitis B virus; RFA, radiofrequency ablation; TACE, transcatheter arterial chemoembolization; α-FP, alpha-fetoprotein.*

^*^Three patients had 2 metastatic sites.

^**^Sixteen patients received ≥2 types of previous treatment.
